# Characterization of the Microbial Population Inhabiting a Solar Saltern Pond of the Odiel Marshlands (SW Spain)

**DOI:** 10.3390/md16090332

**Published:** 2018-09-12

**Authors:** Patricia Gómez-Villegas, Javier Vigara, Rosa León

**Affiliations:** Laboratory of Biochemistry and Molecular Biology, Faculty of Experimental Sciences, Marine International Campus of Excellence (CEIMAR), University of Huelva, 21071 Huelva, Spain; patgomvil@gmail.com (P.G.-V.); vigara@uhu.es (J.V.)

**Keywords:** halo-extremophyles, archaea, 16S rRNA metagenomics, haloenzymes, Odiel marshlands

## Abstract

The solar salterns located in the Odiel marshlands, in southwest Spain, are an excellent example of a hypersaline environment inhabited by microbial populations specialized in thriving under conditions of high salinity, which remains poorly explored. Traditional culture-dependent taxonomic studies have usually under-estimated the biodiversity in saline environments due to the difficulties that many of these species have to grow at laboratory conditions. Here we compare two molecular methods to profile the microbial population present in the Odiel saltern hypersaline water ponds (33% salinity). On the one hand, the construction and characterization of two clone PCR amplified-16S rRNA libraries, and on the other, a high throughput 16S rRNA sequencing approach based on the Illumina MiSeq platform. The results reveal that both methods are comparable for the estimation of major genera, although massive sequencing provides more information about the less abundant ones. The obtained data indicate that *Salinibacter ruber* is the most abundant genus, followed by the archaea genera, *Halorubrum* and *Haloquadratum*. However, more than 100 additional species can be detected by Next Generation Sequencing (NGS). In addition, a preliminary study to test the biotechnological applications of this microbial population, based on its ability to produce and excrete haloenzymes, is shown.

## 1. Introduction

The study of the microbial population inhabiting extreme saline environments has gained increasing interest in the last years due to its usually uncompleted characterization, which is essential to understand the ecology of these ecosystems [1] and also because archaea have revealed themselves as the key to understand the origin of eukaryotic cells [2]. Furthermore, these halo-extremophyles microorganisms can be an excellent source of useful compounds and proteins with special properties and potential industrial applications [3,4] such as antioxidant pigments [5,6], haloestable enzymes [7], antimicrobial compounds [8] or antitumor agents [9]. Haloenzymes have unique characteristics that allow them to be stable and functional at saline concentrations as high as 5 M and tolerate high temperatures without losing their activity [10]. This fact makes halotolerant archaea a potential source of enzymes for food, textile, pharmaceutical or chemical industries [11].

The extreme conditions that prevail in salt brines, which include high light intensity, UV radiation, elevated temperatures and salt concentrations near saturation, support a considerable diversity of halophilic microorganisms belonging mainly to the haloarchaea group [12]. Traditional ecological studies, based on serial dilutions or streaking on agar plates for single-cell isolation, have usually underestimated this biodiversity due to the difficulties of some species to grow at lab conditions. Examples of this are the unsuccessful attempts to culture some generally abundant archaea genera, such as the square-shaped *Haloquadratum* [13]; or the discrepancy commonly found between the characterization of microbial communities by culture-independent and culture-dependent methods.

The application of molecular techniques, based on the comparison of highly conserved DNA reference sequences, such as the genes encoding for ribosomal RNA (16S rRNA, 5S rRNA), has allowed to overcome this limitation, making possible the comparison of different microbial communities and the discovery of a good number of uncultured new species. Examples of these culture-independent methods include: random fragment length polymorphisms (RFLP) [14], fluorescence *in situ* hybridization with rRNA-targeted probes [15], denaturing gradient gel electrophoresis (DGGE) [16] and more recently metagenomic approaches [17,18], which have facilitated the profiling of complex microbial communities. Culture-independent characterization of the microbial assemblages in different halophilic habitats has shown that diversity within the domain archaea is broader than that previously inferred from culture-dependent surveys [19]. 

The solar salterns located in the Odiel Marshlands (Huelva), at the southwest of Spain, are an excellent example of a hypersaline environment inhabited by microbial populations specialized in thriving under conditions of high salinity, which remains poorly explored [20]. In this work, we have used two independent methods to study the prokaryotic diversity present in these salterns. We have constructed and characterized two libraries of PCR amplified 16S rRNA genes obtained using genomic DNA extracted from a water sample of the brines as template; and we have used a high throughput 16S rRNA massive sequencing approach based on the Illumina MiSeq platform to profile the same genomic sample. This double approach has allowed us, not only to explore the microbial diversity of this water environment but also to validate the results of the PCR gene library by comparison with the NGS approach. Furthermore, the ability of this microbial population to produce and excrete haloenzymes with applied interest has been studied.

## 2. Results

### 2.1. Construction of a 16S rRNA Library and Identification of the Obtained Sequences

Two 16S rRNA libraries clone libraries, one for archaea and another one for bacteria, were constructed from an environmental water sample collected at the end of the summer, in the crystallizer ponds located in the Marshlands of the Odiel river in the southwest coast of Spain. The procedure for the libraries construction is detailed in Section 4.4 and the specific primers used listed in Table S1 (Supplementary Material). The main chemical characteristics of the water at the collection time are summarized in Table 1. The salt composition was similar to that reported for other thalassohaline marine solar salterns at this degree of salinity, which was 33.2% at the time of sample collection. 

**Figure 1:**
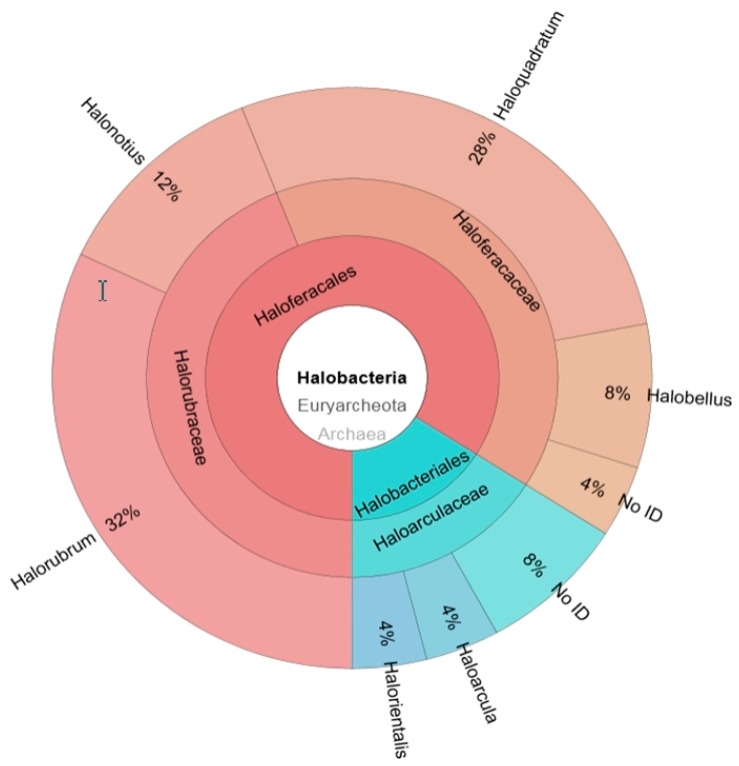
Distribution of the clones from the archaeal 16S rRNA clone library among different genera. 16S rRNA fragments obtained by amplification with archaeal specific primers were cloned into pGEMT vector for the construction of a clone library. The inserted sequence of 25 of the obtained clones were analysed and compared with the NCBI to identify the original genera. Data are expressed as percentages of the total archaeal population. Only sequences that shared over 95% 16S rRNA sequence identity with a known one was assigned to a specific genus.

A selection of 50 clones from both clone libraries, 25 clones per each library, were analysed. The preliminary comparison of the obtained sequences with the National Center for Biotechnology Information database (NCBI) revealed that many of the clones in the 16S rRNA libraries were redundant. In the archaeal library, the 25 clones studied corresponded to 11 different species, belonging to six genera (Figure 1). Most clones corresponded to the archaea genus *Halorubrum*, followed by the peculiar square-shaped haloarchaea *Haloquadratum* [13,21]. These two genera represented respectively 32% and 28% of the total archaeal clones obtained, followed by *Halonotius* (12%) and *Halobellus* (8%). The least abundant genera identified were *Haloarcula* and *Halorientalis*, each one represented 4% of the total clones (Figure 1). Three clones could not be directly affiliated to any currently described genera, since they did not reach the 95% of sequence identity with any of the sequences of the database. The Shannon biodiversity index was calculated as previously reported [22], obtaining a value of 2.07. Despite the small number of clones analysed this score indicates a wide range of diversity.

**Figure 2:**
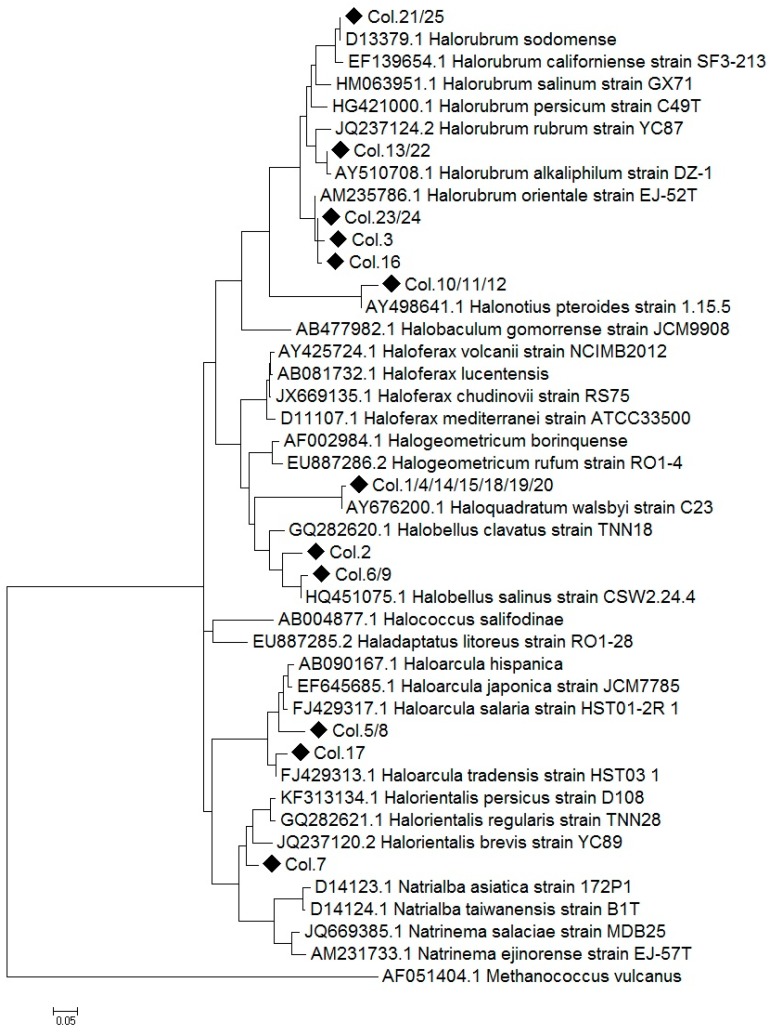
Molecular Phylogenetic Analysis by Maximum Likelihood method. The tree represents the relationship among the 16S rRNA sequences from strains isolated from the saltern ponds of the Odiel Marshlands and reference archaeal sequences. Multiple alignments were generated by MUSCLE and the tree was constructed with MEGA 7, using 1000 bootstrap replicates. The name and the NCBI access number are indicated for all the reference sequences. Black diamonds represent 16S rDNA Sequences from the isolates and “Col. N” denotes the colony number. When an identical sequence was obtained from different colonies it was denoted as “Col. N1/N2.” The tree is drawn to scale, branch lengths represent the number of substitutions per site. Scale bar indicate 5% sequence divergence. The sequence of *Methanococcus vulcanus* was used as the outgroup.

To obtain additional information, a molecular phylogenetic analysis has been done including all the 16S rRNA encoding sequences obtained from the analysis of the archaeal library and several reference sequences obtained from the NCBI data base. The evolutionary history was inferred by using the Maximum Likelihood method based on the General Time Reversible model [23] (Figure 2). 

Some of the amplified 16S rRNA sequences obtained in the archaea library showed 100% of identity or were closely related, showing ≤3% sequence divergence, with species already classified and could be assigned at species level. As it is shown in Figure 2, the sequences Col.10/11/12 and Col. 1/4/14/15/18/19/20 can be assigned to the species *Halonotius pteroides* and *Haloquadratum walsbyi*, respectively. The *Haloquadratum* sequences found (Col.1/14/15/18/19/20) displayed sequence divergences lower than 1% with *H. walsbyi*. By contrast, sequences related to *Halorubrum* genus (Col.21/25, Col.13/22, Col.23/24, Col.3, Col.16), *Halobellus* genus (Col.6/9) and *Haloarcula* genus (Col.17) showed high percentage of identity with several reference species. In addition, sequences clustering within *Halorientalis* genus (Col.7) presented more than 3% divergence with the reference sequences; being impossible their assignation to a particular species in these cases. Finally, two of the sequences which could not be directly assigned to a genus (Col.5, Col.8) clustered within the *Haloarcula* clade, while Col.2 is strongly related to the genus *Hallobelus*.

By contrast, all the clones isolated from the bacterial gene library contained 16S rRNA sequences with 99–100% sequence identity to a unique bacterial species, *Salinibacter ruber*, which is usually present in hypersaline ponds [24]. The chosen primers have been shown to be very specific for each prokaryotic group studied, since archaea sequences have not been obtained in the bacterial library, nor have bacterial sequences been detected in the archaeal library. This specificity makes impossible the use of these primers to create a common bacterial/archaeal library.

### 2.2. Metagenomic Microbial Profiling by High-throughput 16S rRNA Sequencing 

As a second approach, the identification of the microbial population present in the hypersaline water from the Odiel saltern ponds was performed by next-generation sequencing of the 16S rRNA gene, using the Illumina MiSeq platform as detailed in Materials and Methods. The analysis was set up in quadruplicate by two independent sequencing services, Stabvida (SBV) and Life Sequencing (LFS). Bioinformatic processing with the software pipelines described in Materials and Methods allowed us to cluster the obtained reads into a limited number of operational taxonomic units, between 117 and 356, depending on the sequencing reaction and the bioinformatic treatment of the obtained sequences (Table 2). The mean quality of the processed sequences was denoted by the Q scores and the Shannon biodiversity index (*H’*), calculated including the whole prokaryotic population and following previously described procedures [22,25].

**Figure 3:**
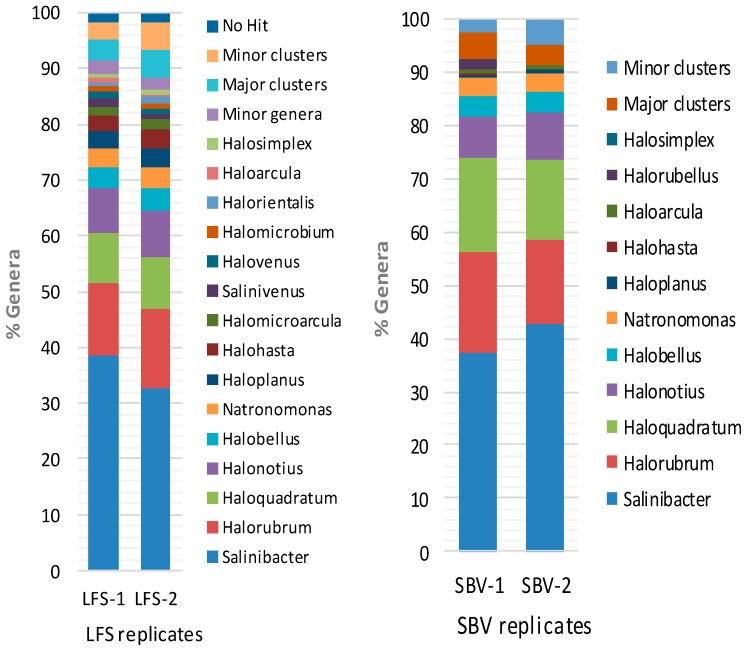
Relative abundance of the genera found by the metagenomic approaches. Operational Taxonomic Units (OTUs) distribution in Odiel saline ponds (33% salinity) obtained from Illumina MiSeq sequencing of the 16S rRNA V3, V4 hypervariable regions. Four data sets were obtained from two different sequencing services. The graphic shows the percentage of the genera with more than 0.2% abundance. Minor genera include all the genera below 0.2%. The sequences that clustered together but could not been affiliated to a genus are named as “clusters” and have been divided in “Major clusters” (>0.2%) and “Minor clusters” (<0.2%). “No hit” represents the sequences which did not cluster with any other obtained sequence.

Both 16S rRNA NGS analysis indicate that the most abundant reads correspond to the halophilic bacteria *Salinibacter* and the archaeal genera *Halorubrum*, *Haloquadratum* and *Halonotius*, although there are significant discrepancies between their relative abundance. *Salinibacter* represents between 38% and 42% of the total reads. *Halorubrum* (13–19%), *Haloquadratum* (9–18%) and *Halonotius* (8–9%) are the main archaeal genera, followed by *Halobellus* (3–4%), *Natronomonas* (2.5–3%). *Haloplanus* and *Halohasta* each represent around 3% of the total sequences after the analysis of Life Sequencing and only trace amounts (0.1%) in the data from Stabvida. *Halomicroarcula*, *Salinivenus*, *Halovenus*, *Halomicrobium*, *Halorientalis*, *Haloarcula* and *Halosimplex*, with relative abundances between 0.7% and 1.5%, are also present in both analyses. Genera with relative abundances lower than 0.2% have not been shown in Figure 3, but the complete list of sequenced genera is shown in Supplementary Material (Table S2).

It is interesting to note that the NGS analysis revealed the presence of trace amounts of genus such as *Spiribacter* (0.1%), a moderate halophilic bacteria usually found in medium salinity habitats [26] and other minor bacteria, not revealed in the clone library approach (Table S2). 

### 2.3. Comparison of Clone Library and 16S rRNA Metagenomic Approaches to Identify the Archaeal Microbiota of the Odiel Saltern Ponds Water

**Figure 4:**
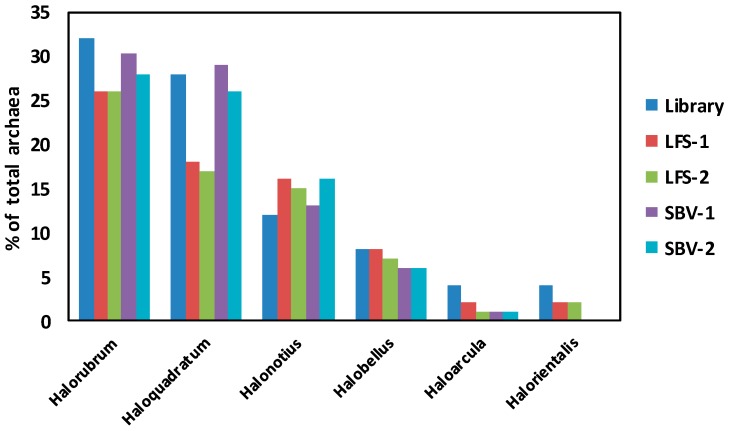
Comparison of the main archaeal genera present in the Odiel saline ponds. Percentage of the different archaea genera over the total archaea population found in the Odiel saline ponds obtained by the two different culture-independent approaches previously described: construction of a clone library (Library) and massive 16S rRNA sequencing, including the two replicates from each Metagenomic Service (LFS and SBV). Only the most abundant genera are shown.

The relative abundances of the main genera obtained from the two NGS platforms are compared with those obtained by affiliation of the 25 sequences gathered from the archaeal clone library (Figure 4). Despite the low number of sequences retrieved from the clone library, there was a considerable degree of agreement between the main genera obtained by this method and by the NGS approaches, as it is supported by a correlation coefficient of 0.97 when comparing clone library results versus the mean of NGS results. Similarly, a correlation coefficient of 0.96 was obtained when we compared both NGS methods (Mean LSF vs. Mean SVB).

The estimated percentage of *Halorubrum* and *Haloquadratum* obtained by the clone library approach are almost identical to those obtained by the Stabvida analysis, being the standard deviation (SD) for these values 2 and 0.34 respectively, while the percentages of *Halonotius* and *Halobellus* are of the same order than those obtained by Life Sequencing (SD 2.48 and 0.35, respectively) or Stabvida (SD 1.78 and 1.41, respectively). *Halorientalis* and *Haloarcula* which represented about 4% of the library clones are also present in the massive 16S rRNA analysis but at lower percentage than that estimated by the clone library approach. Standard deviations in these cases were respectively 1.42 and 1.77, comparing the results of the clone library with the Life Sequencing results; and 2.82 and 2.12 respectively, when comparing with Stabvida results. Massive sequencing provides more information about the less abundant species, although the analysis of higher number of archaeal clones could have allowed the identification of more minor genera by the clone library approach. 

### 2.4. Evaluation of Halocin Activity

**Figure 5:**
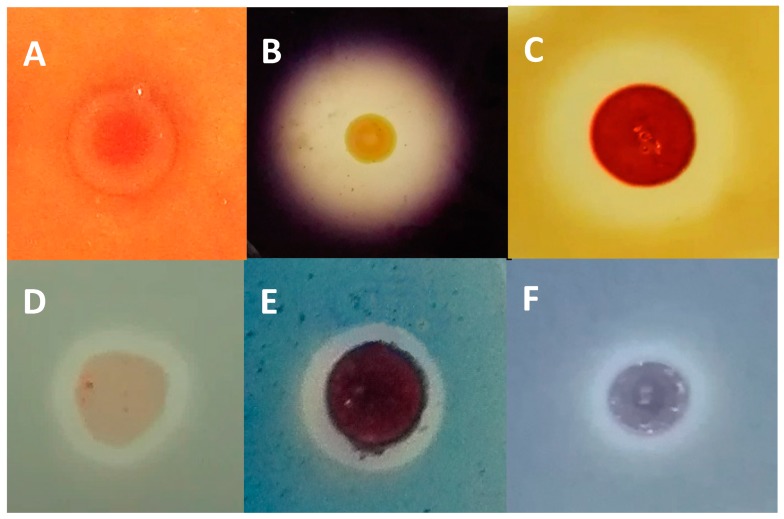
Detection of extracellular halocin and enzymatic activities in the enriched archaeal biomass by plate assay. The biomass obtained from the Odiel salterns water was enriched and concentrated as detailed in Material and Methods and used to test extracellular: halocin (**A**), amylase (**B**), protease (**C**), lipase (**D**), cellulase (**E**) and laccase (**F**) activities by the plate assays described in Materials and Methods.

Massive 16S rRNA sequencing has revealed an extremely low representation of the genus *Haloferax* (0.021%) in the Odiel Saltern ponds (Table S2). However, *Haloferax* is a metabolically versatile genus, able to grow on complex substrates and degrade polymeric substances with a wide salt tolerance in laboratory cultures [27]. To investigate the possible reasons for the low presence of *Haloferax* in the Odiel saltern water, we studied the potential ability of the biomass isolated from the Odiel saterns to specifically inhibit the growth of the control *Haloferax* species, *Haloferax lucetense.* The results show the inhibition of *H. lucetense* growth in the presence of the concentrated biomass isolated from the Odiel saltern ponds, indicating the presence of halocin activity (Figure 5A), which could be an important factor to explain the practically absence of species of the *Haloferax* genus in the hypersaline water from the Odiel saltern ponds. 

### 2.5. Haloenzymes Production by the Archaeal Enriched Biomass Isolated from the Odiel Saltern Ponds

Simple and sensitive plate assays were optimized for the detection of archaeal extracellular enzymes produced by the biomass isolated from the hypersaline water (33%) of the Odiel salterns. The biomass was enriched, concentrated and dropped on agar plates supplemented with different carbon sources to detect the excretion of α-amylase, protease, lipase, cellulase and laccase as described in Materials and Methods.

The amylase (Figure 5B), protease (Figure 5C) and lipase (Figure 5D) activities, assayed as described in Material and Methods following the degradation of starch, skimmed milk and Tween 80, respectively, were positive. Cellulase and exo-lacasse activities, assayed in the presence of carboxymethyl cellulose (Figure 5E) and bromophenol blue (Figure 5F), were also detected. 

## 3. Discussion

### 3.1. Microbiological Diversity in Hypersaline Solar Saltern Ponds 

Despite the existence of many studies about the microbiota inhabiting thalassosaline water ponds all around the word, it is difficult to establish comparisons among them, due to the variety of experimental procedures used to determine microbial composition and the dramatic influence of the water salinity, which can range between the 3.5% of the sea water to the 37% of NaCl saturation.

Numerous studies have described the prokaryotic communities that inhabit saltern crystallizer ponds as distant as the Mediterranean coast of Spain [15,18], Australia [28] or Mexico [29], among others. However, the microbial profile of the Odiel salterns, differently from that of other Spanish salterns, such as Santa Pola in Alicante [24,30] or Isla Cristina in Huelva [18], has been poorly studied. To our knowledge, the only survey about the microbiota of the Odiel Marshlands is a recent study by Vera-Gargallo and Ventosa [20], which focused on studying the assemblage and the metabolic strategies of the microbiota thriving in these hypersaline soils but no information about the aquatic microbial composition of this location has been previously reported. 

Most hypersaline ponds, regardless their location, are dominated by the archaeal genera species *Haloquadratum* and *Halorubrum* [31], which coexists and compete for the same hypersaline environments. *Haloquadratum* was the predominant genus followed by *Halorubrum*, in three crystallizer ponds as geographically distant as Australia at 34% salinity [28], Santa Pola (Spain) at 32% salinity [18] and in Bingöl (Turkey) at 25% salinity [32]. Conversely, our results at 33% salinity suggest that in Odiel Salters this relation is inverse, being the most abundant archaeal genus *Halorubrum* followed by *Haloquadratum*, which often dominates the microbial communities in hypersaline waters and was not cultured in a laboratory until 2004 [13,21]. This difference could be attributed to changes in environmental conditions, due to the diverse geographical situations [18]. There are also some interesting exceptions, such as the Maras salterns in the Peruvian Andes, in which around 31% of the detected sequences were related to the usually low abundant *Halobacterium* and no *Halorubrum* was detected [33]; the Adriatic solar saltern crystallizers studied by Pašić et al. [34], where the presence of the usually abundant *Haloquadratum* was rare; or the Pomorie Salterns (Bulgaria) where the predominant archaea genus was *Halanaeroarchaeum*, which reached 28% of the total archaeal community [35]. The aforementioned study carried out by Vera-Gargallo and Ventosa about saline soils from Odiel saltmarshes reveals that *Haloquadratum* was not found in these soils; in contrast to our results from hypersaline water in the same location, which suggest that this is one of the dominant archaeal genus. However, other genera identified in our work, such as *Halorubrum*, *Haloarcula* and *Halobellus*, are also present in the soil samples studied [20]. The predominant microbial genera and their estimated composition in other hypersaline ponds with salinity similar to our study are summarized in Table 3. The most complete studies have been done in Santa Pola, in the Mediterranean coast of Spain and showed that *Haloquadratum* was the most abundant microorganism and that its relative abundance increased with salinity. In hypersaline ponds in Australia (34%) [28] the three most abundant haloarchaeal genera were the same that we observed in the Odiel ponds. However, it is important to notice that in this study and in the study carried out by Kambourova et al. in Bulgaria [35], the bacterial contribution to the prokaryotic community was not considered because the study was based on clone libraries with archaeal specific primers. This comparison confirms that although there are some common genera, which are found in almost all hypersaline ponds, such as *Halorubrum* or *Haloquadratum*, the relative abundance of hypersaline genera is specific of each geographic location. In addition, identifications at species level returned unique lineages which appeared to be specific of the investigated environments.

*Salinibacter ruber*, was first identified in Santa Pola, Spain (Alicante, Spain) and, despite not being an archaea, it is usually one of the most abundant microorganisms present in hypersaline waters [24]. In Santa Pola ponds with salinities of 19%, 33% and 37% the abundance of *Salinibacter* was reported to be 6.4%, 4.7% and 9.1%, respectively [18]. Our results suggest that the bacteria *Salinibacter ruber* is the dominant microorganism in the Odiel saltern water with a salinity of 33%, reaching around 40% of the total prokaryotic community (Figure 3), which is the highest *Salinibacter* abundance reported in solar salterns to our knowledge. The libraries for the metagenomics studies were built with the universal primers, which have been designed to target V3 and V4 hypervariable regions from both bacterial and archaeal 16S rRNA [36]; however, additional research is needed to determine if the high percentage of reads corresponding to *Salinibacter* obtained in our metagenomic study corresponds to such a percentage of the bacterium abundance or can be influenced by a potential bias in the library construction. 

It is interesting to note the low abundance that we have found for the genus *Haloferax* in the Odiel evaporation pond. Although it grows optimally at 2.5 M NaCl (15% salinity) [37], *Haloferax* has been described to grow at salinities of 33.7% with growth rates higher than any other comparable extreme halophile [27]. Despite these characteristics, *Haloferax* is usually found at low percentage, around 1%, in solar saltern ponds with medium and high salinity (10–37%), as described for example in Santa Pola, Spain [30]. Curiously, the relative abundance that we have found in the Odiel evaporation pond for *Haloferax* is even lower, reaching about 0.021% of the total prokaryotic community. There are several studies that have shown the excretion of halocins or archeocins by archaeal species [38,39], demonstrating the importance of such halocins for interspecies competition in hypersaline environments. The results of our growth inhibitory studies support the possible production of halocins against *Haloferax* by the dominant species and give a possible explanation to the limited presence in the Odiel salterns of *Haloferax*, which should be apparently more qualified to dominate hypersaline ecosystems than the usual dominant species [27].

### 3.2. PCR Library versus 16S rRNA Massive Sequencing

Cloning-based methods have been successfully used for years [40], however the availability of new benchtop NGS technologies has made more popular the use of 16S rRNA high-throughput for sequencing and profiling of microbial communities including that of hypersaline habitats [41]. 

Here we demonstrate that, although NGS 16S rRNA sequencing offers a more complete view of the microbial community inhabiting the saline ponds of the Odiel Marshlands, providing more information about the less abundant genera, both NGS and 16S rRNA clone library approaches are comparable regarding the estimation of the major genera found in the sample. This is in agreement with the results obtained by other authors, such as González-Pimentel [42], who compared both approaches to study the microbial diversity in lava tubes from Canary Islands. 

We have observed that there is a good general agreement in the relative abundance of the main genera distribution obtained in both NGS analysis (SBV and LFS), however there are discrepancies for some genera. Since both massive sequence services have used the same platform (My Seq Illumina), primers and starting genomic DNA, the small discrepancies and the higher sensitivity of LFS analysis can be greatly attributed to differences in the bioinformatic analysis and other factors such as details of PCR libraries preparation. Furthermore, we observed that the use of different tools for the denoising and clustering step (i.e., using DADA2 instead of DEBLUR plugging) caused the removal of potentially valid sequences and yield different results from the same raw data (data not shown).

Shannon index value, which indicates the uniformity of species and its abundance in the obtained OTUs, increases as both the richness and the evenness of the community increase [43]. The Shannon index values calculated for our NGS studies were on average 2.9 for LFS replicates and 2.7 for SVB replicates (Table 2), while the Shannon index calculated for the clone library data was 2.07. This value is in accordance with previous studies carried out by clone library approaches, which reported values between 1.64 and 2.10 in Australia (34% salinity) [28], 1.8 (37% salinity) and 1.6 (38% salinity) in Mexico [29], while Shannon indexes from NGS are substantially higher. This difference is probably due to the small number of sequences analysed by the clone library technique when compared to NGS. It is necessary to remember that both, clone library and 16S rRNA massive sequencing, methods are based on the PCR amplification of a fraction of the highly conserved reference sequence 16S rRNA. Consequently, both methods share the possible bias inherent to PCR amplification of a single gene and the limitations of 16S rRNA for the resolution of closely related species [44]. Some authors have pointed the higher sensitivity and accuracy of whole genome metagenomic sequencing approach [44]. However, the lower cost of the massive sequencing of 16S rRNA and the existence of wide information and databases for the 16S rRNA sequences have converted this approach in the most commonly used method for exploring bacterial communities. It is important to note that to obtain accurate values in the characterization of microbial communities by single gene amplification methods primers must be validated [45], as were the IlluAdp16S primers (Table S1 in Supplementary Material) used in this study [36]; and robust bioinformatic pipelines should be chosen to process the sequencing data, since different bioinformatic treatments usually yield different relative genera abundances.

### 3.3. Archaeal Halo-Exoenzymes

Haloarchaeal hydrolases are halophilic and usually thermostable exoenzymes able to catalyse clean and ecologically-friendly processes with high specificity. They have interesting features which make them very attractive for many industrial applications, such as paper, textile, food, detergent and pharmaceutical industries [46]. In addition, lipases and esterases could be used in biofuel production [47], while cellulases and laccases could be of interest for the conversion of plant biomass into fuel and renewable products [48] and the detoxification of the treated lignocelluloses substrates [49,50], respectively. Despite all these interesting studies no application of archaeal haloenzymes at industrial scale has been described so far [10,51].

Halophilic termoestable α-amylases have been found in different genera, including *Haloferax* [52], *Haloterrigena* [53] and *Halomonas* [54]. Some haloarchaeal isolates, such as *Haloarcula marismortui*, produce salt-dependent thermoactive lipases and esterases [55]. Extracellular organic-solvent tolerant proteases have been found in *Halobacterium* sp. [56] and *Natrialba magadii* [57]. However, only few cellulases [58,59,60] and laccases [61,62] producing halophiles have been reported.

In this work, we have demonstrated that the enriched biomass analysed presents different hydrolases activities, including α-amylase, protease, lipase/esterase, cellulose and laccase. Further studies will focus on the isolation and identification of the strains which show the higher activity for each enzyme, followed by the characterization of the parameters that enable the best activity.

## 4. Materials and Methods 

### 4.1. Sample Collection and Chemical Composition of the Brine

Samples were obtained at the end of the summer from the salt evaporation ponds located in the natural reserve of Odiel Marshlands, at the estuary of the Odiel and Tinto rivers in the Southwest Spain (Latitude: 37.2395, longitude: −6.95287). The salt concentration in the crystallizer pond was 33.2% at the collection time. Climatological features of the location are characteristic of the Mediterranean maritime climate with hot dry summers and rainy autumns and winters. The mean insolation rate exceeds 3000 h per year, the average annual rainfall and air temperature are 506 mm and 18.3 °C, respectively [20]. The ionic composition and the main physicochemical parameters of the seawater brine at the time of sample collection were determined according to following standard methods: ISO 2480-1973 “Determination of sulphate contents-barium sulphate gravimetric method”; ISO 2481-1973 “Determination of halogens expressed as chlorine-mercuric method”, ISO 2482-1973 “Determination of calcium and magnesium contents-EDTA complexometric method”; ECSS/SC 2482-1979 “Determination of potassium content by flame atomic absorption spectrophotometric method”.

### 4.2. Genomic DNA Extraction

For genomic DNA extraction, fresh biomass was harvested by centrifugation of a 500 mL water sample at 11,000 rpm. The resulting pellet was washed with ammonium formate 4 M, freeze-dried and used for genomic DNA extraction with the GeneJET Genomic Purification kit (Thermo Fisher Scientific, Waltham, MA, USA), following the instructions of the manufacturer. Quantification of the genomic DNA obtained and assessment of its purity was done on a Nanodrop Spectrophotometer ND-1000 (Thermo Fisher Scientific).

### 4.3. Amplification of 16S rRNA Encoding Gene and Construction of Clone Libraries

16S rRNA fragments were amplified with the primer sets: Arc340F/Arc1000R [6,63] for archaea and 341F/907R [64,65] for bacteria (Table S1) using 1 µL of genomic DNA, isolated as previously described, as template. Polymerase chain reactions (PCR) were performed in a total volume of 25 µL containing 1 µL of genomic DNA, 10 pM of each primer, 0.2 mM dNTPs, 0.5 U Taq DNA polymerase from Bioline, 2.5 µL of specific 10X buffer and 1.5 µL of 2.5 mM MgCl_2_ buffer using an Eppendorff thermo-cycler. The PCR program was 0.5 min at 96 °C, 0.5 min at 50 °C and 1 min at 72 °C for 30 cycles, followed by 10 min of final primer extension. 

### 4.4. Construction and Analysis of Clone Libraries

The PCR products, obtained with both bacterial and archaeal primer sets, were subjected to agarose electrophoretic separation. The bands obtained for each PCR reaction (with around 660 bp for archaea and 560 bp for bacteria) were purified with the GeneJET Gel Extraction Kit (Thermo Fisher Scientific), ligated to pGEM-T vector (Promega, Madison, WI, USA) according to the manufacturer’s instructions and cloned into *Escherichia coli* DH5α competent cells to establish two clone libraries, one for archaea and another one for bacteria. A selection of 50 clones, 25 per each library, were analysed by extraction of the plasmidic DNA, Sanger-sequencing of the 16S rRNA DNA encoding fragments (Stabvida, Lisbon, Portugal) and comparison of the obtained sequences with the National Center for Biotechnology Information 16S rRNA database (NCBI, http://www.ncbi.nih.gov) by using the advanced BLASTN search tool. Sequences with more than 98% length coverage and more than 95% of sequence identity were assigned to a described genus. Sequences with high identity (>97%) were assigned to specific species. Sequences which showed percentage of identity lower than 95% can be potential novel species or genera but further evidence is needed to confirm it. 

### 4.5. High-Throughput 16S rRNA Sequencing

For High-throughput 16S rRNA based microbial profiling, the same genomic DNA was analysed on the Illumina MiSeq platform. Analysis was set up in quadruplicate by two independent Sequencing services: Life Sequencing (Valencia, Spain) and Stabvida (Lisbon, Portugal). In both cases the PCR libraries were prepared by targeting the V3-V4 hypervariable regions of the 16S rRNA [36] with the previously validated [66] IlluAdp16S primers (Table S1) and sequenced using the Illumina MiSeq Reagent kits, V2× 250bp or V3× 300 bp, following Illumina recommendations for Library preparation and metagenomic sequencing. R1 and R2 reads were overlapped using PEAR program version 0.9.1 [67]. Raw data were processed for denoising, filtering (minimum quality threshold of Q20) and clustering using different approaches. 

Samples sequenced by STABVIDA (SBV) were processed with QIIME2 v2018.02 [68] and Deblur plugin [69]. The resulting sequences were clustered in operational taxonomic Units (OTUs) and taxonomic assignments were done by scikit-learn naïve Bayes machine learning, which was trained using the SILVA database (version 128) with a clustering threshold of 97% similarity. Samples sequenced by Life Sequencing (LFS) were processed with CUTADAPT 1.8.1 [70] and UCHIME [71] programs. The resulting sequences were clustered in operational taxonomic Units (OTUs) with a threshold of 97%. Those clean FASTA files were BLAST [66] against NCBI 16s rRNA database using BLASTN version 2.2.29+. The resulting XML files were processed using a Python script developed by Life sequencing S.L.-ADM (Paterna, Valencia, Spain) in order to annotate each sequence at different phylogenetic levels.

The Q scores, which represent the probability that a base call is erroneous in a logarithmic base and the Shannon biodiversity index (*H’*), which indicates the uniformity of species and its abundance in the obtained OTUs were calculated following previously described procedures [22,25] and included as sequence quality indicators. 

### 4.6. Extracellular Hydrolases Test

Biomass from the saltern water samples was harvested by centrifugation at 11,000 rpm and resuspended in the archaea enrichment medium (ATCC 1176 medium). Typically, 5 L of environmental water were centrifuged to obtain 50 mL of culture, which was incubated at 37 °C and 100 rpm for 7 days. The biomass growth was quantified by measuring the O.D. at 580 nm in a UV–Vis spectrophotometer Ultrospec 3100 pro. Culture media contained (per litre): 10 g Glucose, 156 g NaCl, 13 g MgCl_2_·6H_2_O, 20 g MgSO_4_·7H_2_O, 1 g CaCl_2_·6H_2_O, 4 g KCl, 0.2 g NaHCO_3_, 0.5 g NaBr, 5 g yeast extract. The pH of medium was adjusted to 7 before autoclaving. After 7 days of growth, the culture was collected by centrifugation at 11,000 rpm and the pellet was resuspended (1/100) using the aforementioned medium. This enriched-concentrated archaea mixture was used to detect different hydrolase activities by platting 20 µL drops of the concentrated biomass on 1% agar plates with the indicated medium supplemented with starch, skim milk, carboxymethylcellulose, bromophenol blue and Tween 80 as substrates to test for α-amylase, protease, cellulase, laccase and lipase activities, respectively.

To test for amylase activity, starch (1% *w*/*v*) was added to the glucose-less agar ATCC 1176 medium. After incubation, the plates were flooded with Lugol reagent solution. The presence of a clear zone around the cells indicated starch hydrolysis. The biomass was screened for proteolytic activity by using ATCC 1176 agar medium supplemented with skimmed milk (1% *w*/*v*). Protease activity detection was based on the presence of a clear zone around the cells growth due to casein hydrolysis. Screening of cellulase production was done on ATCC 1176 agar medium containing carboxymetylcellulose (0.5% *w*/*v*) instead of glucose as carbon source. Plates were flooded with 0.1% Congo red dye for 20 min followed by treatment with 1 M NaCl for 15 min and finally with 1 M HCl for 5 min in order to increase the halo contrast as described by Sazci et al. [72]. The presence of clearance zones around the cells, as a result of carboxymetylcellulose hydrolysis, indicated production of cellulase. Laccase activity assay was carried out on agar Petri dishes containing the ATCC 1176 medium supplemented with the dye bromophenol blue (0.02% *w*/*v*), according to Tekere et al. [73]. The formation of discoloration halos around the cells caused by dye degradation showed laccase activity. Lipase activity was screened on nutrient agar plates containing per litre: 10 g peptone, 150 g NaCl, 1 g CaCl_2_·2H_2_O Tween 80 (0.1% *v*/*v*). Opaque halos around the cells resulting from the precipitation of calcium oleate revealed lipase activity [74]. 

All the plates were incubated at 37 °C and the results were checked periodically from the 3th to 10th day of assay, by measuring the diameters of clearance zones or halos around each archaeal drop. All the tests were done in triplicate.

### 4.7. Growth Inhibition Test

Halocin activity was determined by observing growth inhibition of a presumably susceptible archaea strain. The enriched biomass, obtained as described for hydrolase activities, was tested against *Haloferax lucetense* (CECT 5871), which was purchased from CECT (Spanish Collection of Culture Type). *H. lucetense* was grown on the medium specified by the CECT (MHE 25 Medium; CECT 188) and 1 mL of the culture was completely spread across the surface of a Petri dish. When the plate was totally dried, a 20 µL drop of the enriched biomass was spotlessly placed in the centre of the Petri dish. The inhibition of *H. lucetense* growth was measured by the formation of a clearance zone around the enriched biomass drop added. 

## Figures and Tables

**Table 1:** Chemical composition of the water sample collected from the evaporation ponds located in the Natural Reserve of Odiel Marshlands in the southwest Spain.

Density (g·mL^−1^)		Brine Composition (g·L^−1^)					Total Salinity
	CaSO_4_	MgSO_4_	MgCl_2_	NaCl	KCl	NaBr	
1.212	1.40	23.06	34.08	265.38	7.51	0.84	332.30

**Table 2:** Sequence data statistics.

Reaction	Raw Sequence Reads	Mean Read Length (bp)	Sequences after Denoising	Mean Quality (Q Score)	OTUs	Shannon Index
SBV-1	349 726	250	49 100	>28	177	2.75
SBV-2	204 766	250	19 537	>28	117	2.65
LFS-1	57 148	299.8	25 479	37.16	228	2.77
LFS-2	156 520	299.6	71 623	37.25	356	3.03

The number of sequences and Operational Taxonomic Units (OTUs) obtained from Stabvida (SBV) and Life Sequencing (LFS) are shown. The mean quality, expressed as Q scores and the Shannon biodiversity index for each sequencing run have also been included.

**Table 3:** Comparison of prokaryotic diversity at genus level in the Odiel hypersaline pond (33%) and other hypersaline ponds with similar salinity.

Sample	Santa Pola (Spain)	Santa Pola (Spain)	Odiel Salterns (Spain) *	Pomorie (Bulgaria) **	Bajool (Australia) **
Salinity	33%	37%	33%	34%	34%
**Ref.**	[18,30]	[30]	**This study**	[35]	[28]
%	*Haloquadratum* 29.5 *Haloruburm* 23.1 *Natronomonas* 5.7 *Salinibacter* 4.7 *Haloplanus* 3.4	*Haloquadratum* 58 *Salinibacter* 9.1 *Nanosalina* 4.0 *Haloruburm* 3.2 *Nanosalinarum* 1.7 *Halomicrobium* 1	*Salinibacter* 37.8 *Halorubrum* 15.5 *Haloquadratum* 12.8 *Halonotius* 8.3 *Halobellus* 3.8 *Natromonas* 3.4 *Haloplanus* 2 *Halohasta* 1.7 *Halorientalis* 0.96 *Haloarcula* 0.67	*Halanaeroarchaeum* 27.8 *Halorubrum 24* *Halonotius 15.7* *Halobellus 6.5* *Halovenus 6.5* *Natronomonas 2.8*	*Haloquadratum 47* *Halorubrum 17.6* *Halonotius 11.7* *Haloplanus-like 11.7* *Natronomonas 2.9*

* Mean of four 16S rRNA NGS data sets. ** In these studies, only archaea were considered.

## Supplementary Materials

The following are available online at http://www.mdpi.com/1660-3397/16/9/332/s1, Table S1: Sequences of the primers used, Table S2: Complete list of sequenced genera.
