# Supplementary material for: Characterization of the Microbial Population Inhabiting a Solar Saltern Pond of the Odiel Marshlands (SW Spain)
Source: Mar Drugs. 2018 Sep 12;16(9):332. doi: 10.3390/md16090332 (PMC6164061; doi:10.3390/md16090332)
Supplement: Supplementary file 1 [file marinedrugs-16-00332-s001.zip › Sup. Material/Table S1.docx]

Table S1. Sequences of the primers used.

| Target | Name | Sequence | Reference |
| --- | --- | --- | --- |
| Bacterial 16S rRNA | 341F  907R | CCTACGGGAGGCAGCAG  CCGTCAATTCMTTTGAGTTT | Muyzer et al., 1993  Teske et al., 1996 |
| Archaeal 16S rRNA | Arc340F  Arc1000R | CCCTAYGGGGYGCASCAG  GGCCATGCACYWCYTCTC | Grantner 2011  De la Vega et al., 2016 |
| V3-V4 16SrRNA hypervariable regions | IlluAdp16SF  IlluAdp16SR | CCTACGGGNGGCWGCAG  GACTACHVGGGTATCTAATCC | Klindworth et al., 2013 |
